# Influence of lignin content in cellulose pulp on paper durability

**DOI:** 10.1038/s41598-020-77101-2

**Published:** 2020-11-17

**Authors:** Edyta Małachowska, Marcin Dubowik, Piotr Boruszewski, Joanna Łojewska, Piotr Przybysz

**Affiliations:** 1 Institute of Wood Sciences and Furniture, Warsaw University of Life Sciences - SGGW, 159 Nowoursynowska Str., 02-787 Warsaw, Poland; 2 Natural Fibers Advanced Technologies, 42A Blekitna Str., 93-322 Lodz, Poland; 3 Faculty of Chemistry, Jagiellonian University, Gronostajowa 2, 30-387 Kraków, Poland

**Keywords:** Analytical chemistry, Chemical engineering, Materials chemistry

## Abstract

Paper degradation on a macroscopic scale is characterised primarily by yellowing, an increase in brittleness, and other destructive changes caused by the hydrolysis of glycoside bonds and oxidation reactions. Until now, lignin has been believed to cause these changes. However, contemporary analysis has not confirmed this assumption and has attributed low paper resistance to ageing with acidification owing to the production in acid environments that involve aluminium sulfate. In view of the common belief this manuscript presents studies on the accelerated ageing of papers with different lignin contents that are produced in neutral environments. To achieve the objective, artificially aged papers under conditions of increased humidity and temperature were investigated using chromatographic (SEC) and spectroscopic (FTIR and UV–Vis spectroscopy) techniques. Mechanical tests were used to determine the decrease in tensile properties of the samples. We observed no effects of the lignin content on the ageing rate of paper produced at neutral pH. This work also reveals the extent to which spectroscopic methods are useful for studying the papers containing lignin.

## Introduction

The importance of paper as an information carrier is not diminishing, despite the fact that the development of electronic data carriers has reached an immense rate^1–4^. This is caused by the many advantages of paper, including its high durability. High-quality paper that is stored under proper conditions degrades extremely slowly, and the strength and optical properties are preserved for centuries^5–7^. However, these paper properties rapidly reduce if the paper is produced and stored in an unfavourable environment^8,9^.

Regardless of the complexity of the paper material, the two main chemical pathways of degradation are hydrolysis and oxidation^10–12^.

Glycosidic bonds in cellulose ensure its stability only in an alkaline and neutral environment, whereas increasing the concentration of hydronium ions in an acid medium accelerates the process of hydrolytic degradation of glycosidic bonds. This results in shortened cellulose chains, which manifests as a decrease in the degree of polymerisation^13–15^. The mechanism of acidic hydrolysis is well established in the literature^16^.

**Figure 1:**
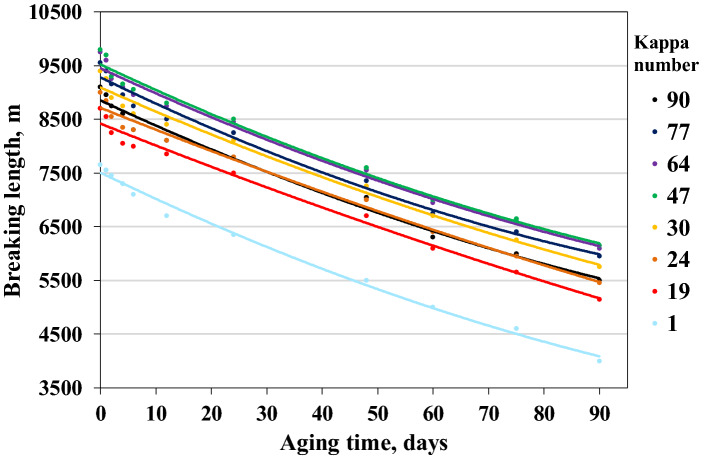
Effect of ageing time on changes in the breaking length of papers with different lignin contents.

While depolymerisation of cellulose through hydrolysis results in a loss of the mechanical strength of paper, oxidation also leads to paper discoloration via the formation of carboxylic groups^17–20^. Oxidation that occurs via a radical mechanism initiated by active oxygen species (O, O2·)^18^ is a much more complex process than hydrolysis (Fig. 1) but is less well described in the literature^7,21^. In acidic and neutral environments, oxidation of cellulose can begin on the hydroxyl groups of the C(2), C(3), and C(6) atoms in a glucopyranose unit. The oxidation initiated through the formation of hydroperoxides proceeds through consecutive and parallel paths^22,23^, leading to the formation of various carbonyl groups, starting from ketones on C(2) and C(3) that become conjugated diketones or starting from aldehydes and to carboxyls on the C(6) carbon atoms of glucopyranose. To a minor extent, active oxygen species may also directly attack the C(1) atom, leading to the oxidative glycosidic bond cleavage. Oxidation (as well as hydrolysis) reactions occur mainly in amorphous regions in cellulose^24^. The hydrolysis and oxidation processes are dependent on each other and catalyse each other.

Lignin is much more vulnerable to oxidation by oxygen in air than cellulose. Thus, papers containing more lignin, hemicelluloses, and additives are generally more sensitive to oxidation (as well as hydrolysis and possible degradation processes), as confirmed by numerous studies^12,25^. Lignin actually plays a two-fold role in cellulose degradation: as an oxidation catalyst (source of radicals) and as an anti-oxidant^26,27^.

Recently, there has been consensus that lignin has an adverse impact on paper durability^12,28^. The reason for this opinion has been the accelerated ageing of paper produced in acid environments and the presence of groundwood pulp. Because of groundwood pulp application, the amount of long fibres in pulp decreases; consequently, paper strength significantly decreases^29^. Conservators have believed that the fibrous component yellows papers because of the high lignin content^30,31^. Moreover, groundwood pulp contains several dozen percent of fine fractions in the form of crushed wood fibres^32,33^. To stop the fine fraction in paper, the application of aluminium sulfate can be initiated. Aluminium sulfate acts as a coagulant that causes the agglomeration and deposition of fine fraction particles on the fibres^34^. The addition of aluminium sulfate also contributes to the increase in retention and effectiveness of glues, fillers, dyes, and auxiliary chemical agents^35,36^. Moreover, it accelerates the drainage of pulp on paper machines, reduces the air content, and decreases the tendency of pulp foaming^37^. It was widely dosed in excess because of these advantages and its low price. For a long time, paper producers did not realise that aluminium sulfate can actually be responsible for the accelerated ageing of paper in the long term.

Beyond the above advantages of using aluminium sulfate, it also causes negative results related to the necessity of paper production in acid environments (pH 4.5–5.5). This results in the acidification of the pulp and the paper produced from it^38^. In some cases, the acidification approaches approximately a pH of 4, resulting in cellulose depolymerisation and a rapid decrease in the tensile properties of paper that has been sized in acid environments^39–41^. Research has shown that the strength of acid paper made from groundwood pulp decreases by 80% after 20 years^42^. Furthermore, the acid environment eliminate the possibility to apply carbonate fillers (CaCO3), which are relatively cheap and easily accessible.

In the second half of the twentieth century, when the mechanism of paper ageing was further investigated, the negative consequences of aluminium sulfate application were realised. The problem of acid paper was called “a chemical disaster” by Richard David Smith^43^. However, aluminium sulfate was difficult to eliminate from the production process because the chemical industry did not produce other auxiliary agents for papermaking. Synthetic coagulants (polyacrylamides, polyethylenimines, polyamidoamines, and others)^44^ that work effectively in neutral environments, were introduced at the end of the 1990s. This allowed exclusion of aluminium sulfate from the paper production process, and paper with increased durability was produced.

Contemporary analysis calls into question the negative impact of lignin on paper durability^45,46^. Canadian research indicated that the presence of lignin has no disadvantage for the stability of the chemical and mechanical paper properties. The role of acidification in accelerated ageing of paper has been decisive^47–49^. Researchers questioned the general conviction that the application of groundwood pulp rich in lignin is one of the major reasons for the low strength of nineteenth and twentieth century paper^50^.

The fact that the acidity of paper is the most important reason for a decrease in tensile properties during natural ageing was confirmed by research results conducted by William James Barrow in the 1950s^51^. However, there is currently no clear division of this topic. One claim that acidic pH affects the rapid progress of paper ageing is challenged by others who suggest that the acidic pH of paper is caused by the presence of lignin in the cellulose fibres. This fact has led authors to undertake studies in this direction. A literature review has shown that no information is available on the impact of lignin content on the ageing of paper produced in neutral environments (without aluminium sulfate). This scientific question is even more important for economic reasons because the market trend is to increase the use of different pulps with higher lignin contents in graphic paper production, e.g. chemi-thermomechanical pulp (CTMP), bleached chemi-thermomechanical pulp (BCTMP), thermomechanical pulp (TMP), and wastepaper.

## Materials and methods

### Delignification process

Pine wood (*Pinus sylvestris* L.) was used in this work. Cellulosic pine pulps were prepared using the sulphate method described by Modrzejewski et al.^52^ from industrial woodchips containing 7–8% moisture. The materials were kept in a hermetically sealed container to avoid any changes to their humidity before treatment with NaOH and Na2S solutions, which were prepared fresh before use. 20–38% active alkali was added (per batch) and the water to wood ratio (v:w) was 4. The dry weight (DW) of all materials was determined before pulping.

The delignification processes were conducted in 15 dm^3^ PD-114 stainless steel reactors (Danex, Katowice, Poland) with regulated temperature (using a water jacket) and agitation (three swings per minute, 60° swing angle). Suspensions of the disintegrated materials were heated for 120 min to achieve a temperature of 172 °C and incubated at this temperature for a further 120 min. The temperature was then decreased to 25 ± 5 °C using a jacket with cold tap water. After delignification, the material was washed several times with demineralised water and incubated overnight in demineralised water to remove the residual alkali-soluble fractions. The solids were disintegrated for 3 min in a laboratory JAC SHPD28D propeller pulp disintegrator (Danex, Katowice, Poland), and the fibres were screened using a PS-114 membrane screener (Danex, Katowice, Poland) equipped with a 0.2 mm gap screen. After screening, the pulps and shives were dried at room temperature (20 ± 2 °C) for 48 h and then weighted to determine the pulp and shive content. The dry pulps were stored in hermetically sealed vials until being used in further experiments. The residual lignin content expressed as the Kappa number (ISO 302:2015) of the pulps was determined. Pulping process advances and method descriptions that are presented in this section were described in our earlier published research^53–55^.

### Bleaching of pulps

In order to really assess the influence of lignin on the depolymerisation of the cellulose, a completely bleached pine pulp sample was also examined for comparison reasons.

The peroxide bleaching phase was conducted using bleach liquor consisting of: 3% H_2_O_2_, 2.5% NaOH, 0.2% MgSO_4_ and 1% Na_2_SiO_3_ (o.d. basis). The pulp (20 g o.d.) and the bleaching reagents were blended with distilled water in a glass bottle to obtain a final consistency of 10%, immersed in a water bath (LaboPlay, Bytom, Poland), held at 80 °C for three hours and manually mixed at 30 min intervals. At the end of the treatment, the pulp was discharged, filtered, washed with four aliquots of 1000 ml distilled warm water, dried and stored in a plastic bag for further treatment.

### Chemical analysis of pulps

Analysis of the chemical composition of cellulosic pulps included quantification of reducing sugars, lignin, extractives, and ash. Hydrolysates of the tested samples were analyzed using a high-performance liquid chromatography (HPLC) in order to determine the content of glucose and other reducing sugars. The tests were carried out using an Ultimata 3000 chromatograph (Dionex). Optimal conditions for the separation of sugars were obtained using a Rezex RPM—Monosaccharide Pb^2+^ chromatography New Column (bed thickness: 8 µm; dimensions: 7.8 × 300 mm). Mobile phase was water of HPLC purity (100%). The column temperature was 80 °C. Individual sugars content in the eluate from column was determined using a Shodex-RI-10 detector.

Acid insoluble lignin was determined according to the Tappi T222 standard (Acid-Insoluble Lignin in Wood and Pulp). The content of acetone-extractives was defined according to the Tappi T204 standard (Solvent Extractives of Wood and Pulp). Ash content was determined according to the Tappi T211 standard (Ash in Wood, Pulp, Paper, and Paperboard—Combustion at 525 °C). Chemical analysis were performed in triplicate for each pulp.

### Paper sheets

The pulps were used to prepare laboratory test sheets. Before processing, the pulp was soaked in water for 24 h. For this purpose, cellulosic pulps were treated in a laboratory propeller pulp disintegrator JAC SHPD28D (Danex, Katowice, Poland), according to PN EN ISO 5263-1 (2006) at 23,000 revolutions. Cellulosic pulps were refined to 30°SR. The Schopper–Riegler freeness was measured using a Schopper–Riegler apparatus (Danex, Katowice, Poland), according to PN-EN ISO 5267-1 (2002). The refining process was performed in a JAC PFID12X PFI mill (Danex, Katowice, Poland) in which a single batch of 22.5 g of dry pulp was used, according to PN-EN ISO 5264-2 (2011). The next step was forming sheets of paper in a Rapid–Koethen class apparatus. The formation of paper sheets was performed in accordance with PN-EN ISO 5269-2 (2007). Each laboratory paper sheet was described by a basis weight of 80 g/m^2^. Only sheets with a basis weight that ranged from 79 to 81 g/m^2^ were accepted for further investigation. The original source of the method descriptions are our earlier published research^56,57^.

### Artificial ageing tests

Samples of obtained papers with different delignification degrees were tested using the WET90 accelerated ageing test, which is often used and has been described in the literature^12^. The accelerated ageing conditions were adjusted to achieve a maximum effect. The test was conducted in the climatic chamber (Memmert HCP246), according to ASTM D6819 (2007) at elevated temperature and presence of water vapour (90 °C and 59% relative humidity, denoted as “WET” method). The reactor and the conditions were chosen to determine the influence of moisture (climatic chamber) on the sample during degradation. During the ageing time in the chamber, the degradation products diffused to the large volume. The time of sample ageing was 1–90 days.

### Mechanical tests

The test sheets were conditioned for 24 h at 23 °C ± 1 °C and relative humidity of 50 ± 2% in accordance with ISO 187 (1990). The properties of the paper sheets were examined as follows. Breaking length was measured in accordance with PN-EN ISO 1924-2 (2010). The measurements of tear resistance and bursting strength were carried out in accordance with ISO 1974 (2012), and PN-EN ISO 2758 (2014-10E), respectively. The tensile properties were performed for aged and non-aged samples. The original source of the method descriptions are our earlier published research^57^.

### Size exclusion chromatography

Cellulose size exclusion chromatography (SEC) analyses were performed using the derivatives of the original samples, cellulose tricarbanilate (CTC) that was soluble in tetrahydrofuran (THF). The derivatives were prepared according to the procedure described by Stol et al.^58^. Two CTC solutions were prepared for each paper sample and subjected to further analysis. Prior to SEC/UV/MALLS analyses, the CTC solutions were diluted to 3:10 with THF and filtered using 0.45 µL PTFE syringe filters. The average molar mass (MW) and molar mass distribution were determined using a Waters chromatographic system, which consists of an isocratic pump, autosampler, column oven, UV–Vis detector (set at a wavelength of 254 nm) and MALLS detector (Dawn Heleos, Wyatt Technology, working at 658 nm). For separation of the CTC samples, a set of two 25 × 1 cm mixed bed polydivinylbenzene columns were used (Jordi). They were thermostated at 35 °C and proceeded by a guard column (Waters). THF (HPLC grade) was used as the eluent with a flow rate of 1.0 cm^3^/min. The SEC system was calibrated using 11 polystyrene standards of known molecular masses with narrow distributions (Fluka). The procedure and method description of the paragraph are taken from the expert literature^12^.

Both the average molecular weights and CTC molecular weight distributions were calculated based on Zimm’s model, which has been recommended for the average size of macromolecules^59^. It involves plotting the relationship between k·c/R_θ and sin2(θ/2) and matching the straight line that runs across the beginning of the coordinate system. The intersection of the straight line with the ordinate axis, i.e. the determined factor, is the reciprocal of the weight average molecular weight (M_w_). The average CTC molecular weights and molecular weight distributions were converted to cellulose assuming that the degree of cellulose substitution with phenylcarbamoyl groups is 2.925^60^. Subsequently, the average degrees of polymerisation (DP) were calculated based on Eq. (1):

1 $$DP = \frac{{M_{w} }}{{M_{m} }}$$

where *M*_w_—weight average molecular mass.

2 $$\overline{{M}_{w}}= \frac{\sum {w}_{i} {M}_{i}}{\sum {w}_{i}}= \frac{\sum {n}_{i} {M}_{i}^{2}}{\sum {n}_{i}{M}_{i}}$$

M_m_—molecular weight of the substituted glucose unit in the cellulose sequence, which is 510 g/mol.

The sources of the method description were taken from expert literature^12,61^. The measuring system used and the calibration of the measurements are also described in detail in the literature^61^.

### Fourier transform infrared spectroscopy

Diffuse reflectance infrared Fourier transform (DRIFT) spectra were collected on a THERMO/Nicolet 5700 spectrometer with an MCT/A detector equipped with a Harrick Praying Mantis instrument. A sample (diameter of 5 mm) was placed inside the DRIFT instrument, which was continuously purged with dried helium (ca. 15 cm^3^/min). To remove water, the temperature of the appliance was set to 110 °C for 10 s to desorb water from the paper. Prior to the measurements, the temperature was decreased to 30 °C, and the spectra were collected. The desorption progress was followed using the disappearance of the 1650 cm^−1^ band. Overall, 128 scans were collected for each spectrum. For the sake of comparison between various samples, the spectra were normalised using the internal standard method (integral of the band between 2784 and 3016 cm^−1^) and presented as the standardised absorbance (Astd). The degradation progress was traced in the range of 1500 and 1900 cm^−1^ where the carbonyl groups evolve. The procedure and method description of the analysis are taken from the expert literature^12,22,62^.

### UV–Vis spectroscopy

The absorption spectra were measured using a setup from AvantesBV (The Netherlands). The presented spectra resolution was set to 2.4 nm. All spectra were obtained in reflectance units and then recalculated as the Kubelka–Munk absorbance (AKM). Each spectrum was measured in the range of 248 to 1050 nm with a resolution of 2.4 nm. Reflectance measurements were performed for each sample twice. White (Rwb) and black (Rbb) spectra were also measured, and all recorded spectra were normalized using the white standard. Using the original Kubelka–Munk theory, the spectra for each sample measured in the UV–Vis range were converted by means of equations into the reflectance spectra R_ of infinite thickness. A description of the UV–Vis methodology is available in the literature^63,64^.

## Results and Discussion

### Characterisation of pine cellulosic pulps

The pine pulps used to produce the papers for ageing differed in chemical composition. The percentage contents of reducing sugars, lignin, extractives, and ash in the pulps are presented in Table 1. The pulps were also characterized in terms of the yield from the digester and the yield after screening. The percentage of lignin was the highest in the pulp digested with 20% addition of active alkali. The unbleached pine pulps contained from 0.4 to 1.4% extractives. The ash content in the pulps was at a constant level (approx. 1.4%), regardless of the cooking conditions (excl. bleached pulp).

**Table 1:** Chemical composition and properties of the pine cellulosic pulps.

Active alkali	Kappa number	Pulp yield from digester	Pulp yield after screening	Glucose	Mannose	Lignin	Extractives	Ash	DP
[%]	[–]	[–]	[–]	[%]	[%]	[%]	[%]	[%]	[–]
20	89.7 (0.4)	49.33 (0.47)	47.19 (0.55)	79.1 (0.2)	6.0 (0.3)	13.5 (0.2)	1.4 (< 0.1)	1.4 (0.2)	2387 (11)
21	76.5 (0.1)	47.62 (0.39)	46.94 (0.41)	81.2 (0.4)	5.9 (0.4)	11.5 (0.2)	1.0 (< 0.1)	1.4 (0.1)	2414 (17)
22	63.8 (0.4)	46.14 (0.32)	45.68 (0.29)	82.9 (0.4)	6.1 (0.5)	9.6 (0.1)	0.8 (< 0.1)	1.4 (0.2)	2788 (42)
24	46.6 (0.5)	43.64 (0.42)	43.52 (0.35)	85.4 (0.6)	6.2 (0.3)	7.0 (0.1)	0.7 (< 0.1)	1.4 (0.1)	3273 (12)
30	29.6 (0.6)	40.53 (0.36)	40.50 (0.31)	87.5 (0.5)	6.7 (0.3)	4.4 (0.1)	0.6 (< 0.1)	1.4 (0.1)	2282 (40)
34	23.7 (0.4)	38.54 (0.29)	38.54 (0.44)	88.7 (0.7)	6.3 (0.1)	3.6 (< 0.1)	0.5 (< 0.1)	1.4 (0.1)	2053 (4)
38	19.1 (0.6)	36.91 (0.22)	36.91 (0.28)	88.9 (0.4)	6.8 (0.2)	2.9 (< 0.1)	0.4 (< 0.1)	1.4 (0.2)	1589 (35)
38 (bleached pulp)	0.5 (0.1)	37.32 (0.24)	37.32 (0.24)	97.1 (0.5)	2.0 (< 0.1)	0.1 (0.0)	0.0 (0.0)	0.8 (< 0.1)	1074 (7)

The degree of polymerisation of cellulose varied from 1074 to 3273 for the pulps from pine with an active alkali addition of 20–38%. The pulp with a Kappa number of 47 was characterised by the highest DP value; thus, the highest strength parameters for paper from this pulp were expected (Tab. 1). Detailed analysis of the pulp used is presented in our previously published work^65^.

**Figure 2:**
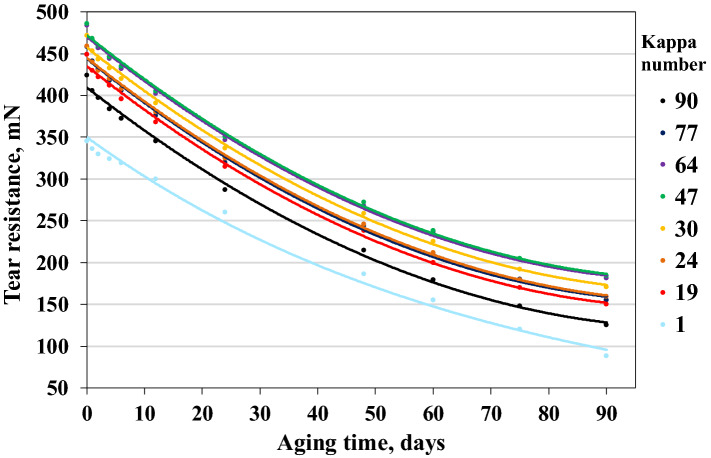
Effect of ageing time on changes in the tear resistance of papers with different lignin contents.

**Figure 3:**
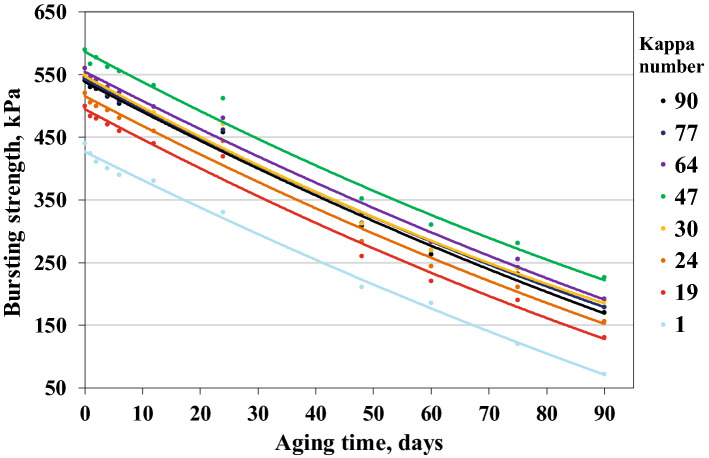
Effect of ageing time on changes in the bursting strength of papers with different lignin contents.

During the alkaline digestion, the polysaccharides were gradually degraded, and then some of them dissolved in the alkaline sulfate solution. Because of the increasing active alkali amount, the destruction and dissolution of hemicelluloses and cellulose increased simultaneously. This reduced the mechanical strength of the fibres (Figs. 1, 2, 3).

### Impact of accelerated ageing on paper strength properties

Mechanical tests performed on the aged samples were used to illustrate changes in the most important strength properties of paper, i.e. breaking length, tear resistance, and bursting strength. The mechanical properties of the unaged and aged papers with different lignin contents are shown in Figs. 1, 2, 3.

The breaking lengths of the examined papers decreased by 37.2–40.8% for unbleached papers and by 47.7% for bleached sample (after 90 days of testing) under WET90 method conditions. The indicator decreased by 3600 m for papers with the highest lignin content and 3650 m for bleached paper with the lowest lignin content (Fig. 1). The results indicate that the amount of lignin in cellulose pulp had an insignificant influence on the amount of decrease in breaking length when an accelerated ageing test was performed, while the initial parameter value depended on the delignification degree of the pulp.

This is especially noticeable in the case of a bleached paper, for which difference to the unbleached paper is 1050 m.

Regarding the tear resistance, the unbleached papers lost 62.1–70.5% of their initial resistance. It decreased by approximately 300 mN, both for paper with the highest Kappa number and for unbleached paper with the lowest Kappa number (Fig. 2). In the case of samples produced from bleached pulp, the final value of tear resistance was lower by 74.5% than the initial value. The initial tear resistance, as for other strength properties, depended on the degree of pulp delignification. However, the amount of lignin content in pulp had no significant effect on the amount of the index decrease under the conditions of this test. The lignin content had a significant impact on the initial tear resistance because the polymerisation degree of cellulose decreased during the digestion process. This resulted in a decrease in the initial value of tear resistance. The results obtained during the SEC analysis confirmed this result (Fig. 5). The nature of the changes in tear resistance as a function of time under accelerated ageing conditions was constant, as in the case of breaking length and bursting strength.

The decrease in the bursting strength of unbleached papers varied from 61.7 to 74.0%. This parameter decreased by about 370 kPa, both for paper with the highest lignin content and for paper with the lowest lignin content (Fig. 3). However, the decrease for bleached papers was the largest (− 83.6%).The results showed that the lignin content in pulp had very little impact on the amount of bursting strength decrease (under WET90 method conditions), even though it defined the initial value.

The results of strength tests for the aged papers were easy to apply to changes in their polymerisation degree (Fig. 5), and these results confirmed the thesis that the main effect of lowering cellulose DP was reduced paper resistance to tearing and increased fragility.

### Impact of accelerated ageing on the depolymerisation process of cellulose

The SEC method allows tracking reactions related to breaking of glycosidic bonds by measuring the average degree of polymerisation and aids in determining cellulose molecular weight distributions. For lignin-containing papers for which the viscometry method fails, and thus for the majority of real archival objects, SEC is the only method available.

**Figure 4:**
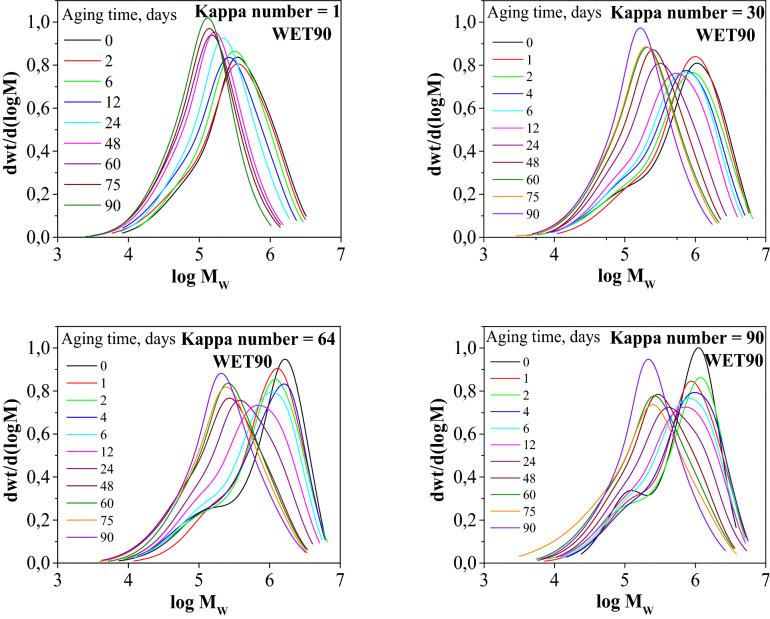
Comparison of the molecular weight distributions of paper samples with different lignin contents.

The distributions of the molecular masses are presented as the differential (density) function of the logarithm from the molecular mass for clarity of the chromatograms. Lignin did not derivatise to trifenylokarbaminian, and it was removed from the samples at the preparation stage. However, hemicelluloses undergo a substitution reaction with phenylcarbamoyl groups. Thus, an additional arm appeared on the chromatograms with increased elution volumes (lower log M_w_ value) as well as an additional maximum on the molecular weight distribution curved line (Fig. 4). The relative intensity of this maximum increased as the lignin content in the tested samples increased (Fig. 4).

Figure 4 show the average molecular weight values from the chromatograms and molecular weight distribution curves for selected tested paper samples with different Kappa numbers (reference and artificially aged). The graphs show significant changes in the shape and location of the molecular weight distributions of samples aged under conditions of increased relative humidity (Fig. 4).

**Figure 5:**
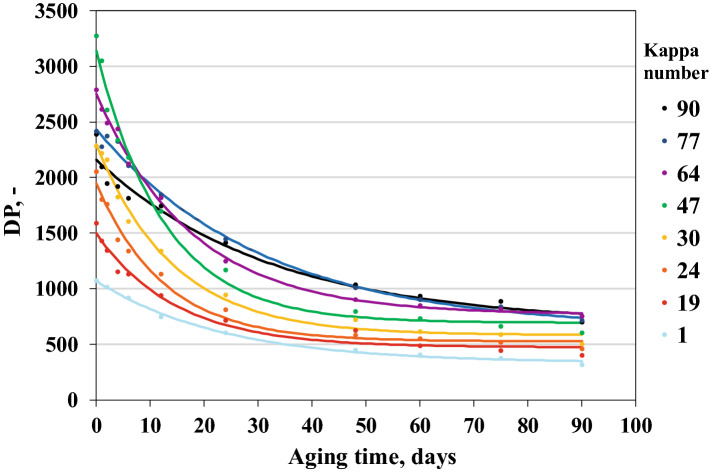
Effect of ageing time on changes in the cellulose DP of papers with different lignin contents that were aged using the WET90 method.

Observation of the progress of cellulose depolymerisation in various samples and under different paper ageing conditions does not require molecular weight distribution analysis that includes a considerable amount of information. The number describing the average weight of cellulose molecules can be used for this purpose. Thus, the obtained molecular weight distributions were used to determine the weight average polymerisation degree (DP). The largest decreases in the DP were observed in the initial ageing period, the dynamics of the DP decreased at a later stage in the tests (after approximately 30 days). The decrease in DP of all papers (irrespective of the lignin content) after the maximum time that the samples stayed in the increased humidity conditions was similar and ranged from 70.4 to 81.5% (Fig. 5). The decrease in cellulose DP under higher humidity conditions was related to the role of water in the cellulose depolymerisation process. The research results indicate that the lignin content in pulp has no significant impact on changes in DP of cellulose under natural ageing conditions. However, the analysis made demonstrates that, an increased lignin content could protect cellulose from depolymerisation. Considering that water molecules could be sources of radicals, the polyphenols, present in lignin, most probably may have captured the radicals, and, therefore, reducing the number of glycosidic bond breakages in cellulose.

### Impact of accelerated ageing on the oxidation process of cellulose

The oxidation progress can be followed by instrumental methods, such as in situ Fourier transform infrared spectroscopy and UV–Vis spectroscopy.

**Figure 6:**
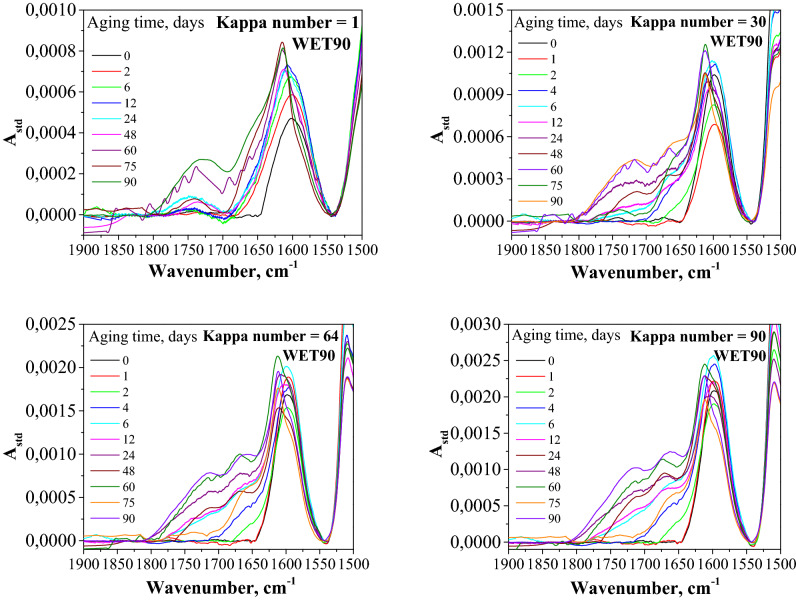
Comparison of transmission FTIR spectra of paper samples with different lignin contents.

Fourier transform infrared spectroscopy (FTIR) is a functional and most commonly used tool for analysis of carbonyl compounds in cellulose^22,66^. Hence, it was used to define the increase in carbonyl groups and thus to investigate the oxidation effects in the tested paper samples. The changes in the oxidation state of the samples with propagating degradation were observed in the FTIR spectra in the range of 1500–1850 cm^−1^. The following trend was noticeable for non-aged samples: the intensity of the band at 1595 cm^−1^ increased as the content of lignin increased. For the wavenumber value of 1740 cm^−1^, the band was observed only for the bleached sample with Kappa number 1 (Fig. 6). It is probably due to a bleaching process which could lead to oxidation of cellulose and lignin. The spectra show more complex progress in the case of artificially aged samples. The carbonyl groups in the cellulose were most formed in the samples with a lower lignin content. In the case of papers with a higher Kappa number, in this region, bands from cellulose oxidation products were expected as well as those from lignin, hemicelluloses, phenolic alcohols of lignin (the p-coumaryl, coniferyl, and sinapyl alcohols), and the chinone derivative. The spectra of samples with a higher lignin content were therefore more complex. With ageing time, the evolution of new bands from lignin was observed at 1712 cm^−1^ and 1661 cm^−1^, which were in the same region as the ν(C=O) bands of carbonyl groups that formed because of cellulose oxidation.

Oxidation of lignin is faster than cellulose oxidation, but like in cellulose, this results in the formation of new carbonyl groups. Thus, changes in the IR spectra that occur because of degradation in the region of the ν(C=O) bands will be related to the oxidation of both polymers. Thus, for the samples containing high lignin contents, Fourier transform infrared spectroscopy does not allow for formulating simple quantitative conclusions, despite the fact that this technique allows us to follow the progress of cellulose oxidation by observing the evolution of the ν(C=O) bands. This is because more reagents undergo parallel, consequential reactions. Furthermore, because there are no individual features in the FTIR spectra that can be ascribed to a specific change in the structure of the tested papers, the method may not be sensitive enough to describe the oxidation progress in lignocellulosic materials.

Figure 6 presents FTIR spectra of selected samples of paper.

UV–Vis spectroscopy, like FTIR, provides observation of carbonyl group formation, but it is not used on a wide scale for this purpose. One problem for the analysis is that there are not many characteristic spectra of non-oxidised and oxidised papers in which there are clearly developed bands.

**Figure 7:**
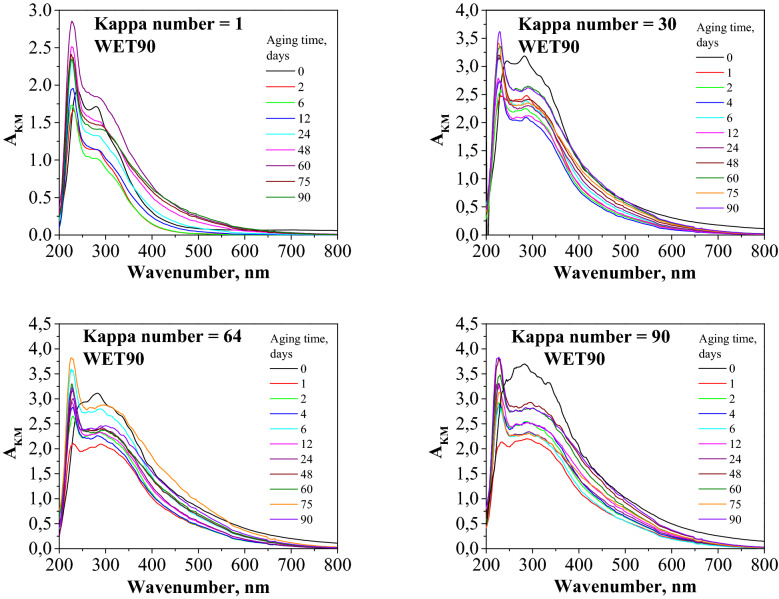
Degradation progress observed according to the changes in the UV–Vis spectra of paper samples with different lignin contents.

In the spectra of the tested papers, transition bands for the π* ← π carbonyl groups with maxima at 289 nm (ketone C(3)), 296 nm (2-enol), 300 nm (ketone C(2)), 339 nm (aldehyde), and 475 nm (diketone) can be observed (Fig. 7).

UV–Vis measurements of paper may include systematic errors because paper samples contain other substances that absorb radiation in the range of 248–1050 nm (in this case, lignin but also phenolic alcohols from hemicelluloses, coloured substances, or some fillers) that can significantly change the spectrum. Additionally, the lignin content makes it considerably more difficult to analyse UV–Vis results. The great disadvantage of the UV–Vis technique is the deficiency of sensitiveness (outside the UV range) to the presence of one of the main products of cellulose oxidation, i.e. carboxyl groups^67^. Therefore, UV–Vis spectroscopy, outside the scope of UV, shows zero sensitiveness to the main cellulose oxidation products, carboxyl groups.

## Conclusions

1. The lignin content in pulp has no significant impact on the ageing rate of paper produced under neutral pH. The decrease in the initial values of the tensile properties after ageing tests was similar, regardless of the Kappa number of the samples. This is a pioneering result in the field of ageing paper research, which is supported by studies using instrumental techniques and mechanical tests. Interestingly, a high concentration of lignin in the paper could even suppress some of the degradation pathways because of the polyphenols, which acted as radical scavengers.
2. A higher temperature decreases the strength properties of paper, which is an important contribution.
3. The increased relative humidity contributes to accelerated cellulose depolymerisation, causing a decrease in the paper strength properties.
4. Papers with a broad spectrum of lignin content could be studied in a non-destructive (UV–Vis, FTIR) or microdestructive (SEC) methods. All the techniques afford complementary information and showed a complex view of the structural changes in lignocellulosic materials in a wide range of Kappa numbers, even though these techniques are more useful for describing the progress of degradation of lignin-free papers.

## Data Availability

The datasets generated during and/or analysed during the current study are available from the corresponding author on reasonable request.
